# An integrated approach to explore the suitability of nitrate-contaminated groundwater for drinking purposes in a semiarid region of India

**DOI:** 10.1007/s10653-022-01237-5

**Published:** 2022-03-10

**Authors:** Balamurugan Panneerselvam, Kirubakaran Muniraj, Karunanidhi Duraisamy, Chaitanya Pande, Shankar Karuppannan, Maciej Thomas

**Affiliations:** 1 Department of Civil, Building and Environmental Engineering, University of Naples Federico II, Naples, Italy; 2 Srii Vickiy and Co, Erode, Tamilnadu India; 3 Department of Civil Engineering, Sri Shakthi Institute of Engineering and Technology, Coimbatore, India; 4 Mahatma Phule Krishi Vidyapeeth Rahuri, Rahuri Ahmednagar, India; 5 Department of Applied Geology, School of Applied Natural Science, Adama Science and Technology University, Adama, Ethiopia; 6 Faculty of Environmental Engineering and Energy, Cracow University of Technology, Cracow, Poland

**Keywords:** Groundwater geochemistry, Nitrate contamination, Risk assessment, GIS techniques, Statistical approach

## Abstract

The main objective of the present study is to perform risk assessment of groundwater contaminated by nitrate (NO_3_^−^) and evaluate the suitability of groundwater for domestic purposes in the Palani region of South India. Thirty groundwater samples were collected in the study area. Various groundwater quality analysis parameters such as the pH, electrical conductivity, total dissolved solids, total hardness, major cations (Ca^2+^, Mg^2+^, Na^+^, and K^+^), and major anions (Cl^−^, SO_4_^2−^, F^−^, CO_3_^2−^, and HCO_3_^−^) were adopted in this study to evaluate the drinking water suitability according to 2011 World Health Organization (WHO) standards. Piper and Gibbs’s diagrams for the tested groundwater indicated that, due to the influence of rock–water interactions, evaporation, and reverse ion exchange, the chemical composition of groundwater varied. According to water quality index (WQI) mapping results, 46.67% of the sample locations was identified as contaminated zones via GIS spatial analysis. Multivariate statistical analysis methods, such as principal component analysis, cluster analysis, and the Pearson correlation matrix, were applied to better understand the relationship between water quality parameters. The results demonstrated that 40% of the samples could be identified as highly affected zones in the study region due to a high nitrate concentration. The noncarcinogenic health risks among men, women, and children reached 40, 50, and 53%, respectively. The results illustrated that children and women occurred at a higher risk than did men in the study region. The major sources of contamination included discharge from households, uncovered septic tanks, leachate from waste dump sites, and excess utilization of fertilizers in the agricultural sector. Furthermore, using the nitrate health hazard integrated method with the conventional indexing approach ensures that groundwater reliability can be guaranteed, contamination can be explored, and appropriate remedial measures can be implemented.

## Introduction

Groundwater contamination has become an environmental issue worldwide. It is necessary to conduct hydrochemical quality assessment of groundwater for multipurpose consumption, such as domestic, industrial, and agricultural uses. Water plays a vital role in the origin, development, and livelihood of every living organism on Earth, which is why most human civilizations have prospered near water (Kumar & Balamurugan, 2019). Groundwater is becoming an essential water source in many parts globally due to its economic and health benefits. Approximately 2.5 billion people rely only on groundwater resources to meet their domestic needs (Jenifer & Jha, 2018). Groundwater is the most critical water source for agriculture and domestic and industrial activities (Kirubakaran et al., 2016). Currently, the demand for water in India has tremendously increased due to the development of various sectors, such as agriculture and manufacturing. As such, groundwater resources have been increasingly considered to meet this demand. In recent years, the demand for freshwater has increased worldwide (Colins et al., 2016). Excessive groundwater utilization has adversely affected the water quality. A lack of subsurface hydrological understanding could further deteriorate the groundwater quality. Approximately 80% of health issues and diseases worldwide can be attributed to the consumption of contaminated water for domestic purposes (Karunanidhi et al., 2019). Groundwater quality assessment could facilitate the development of groundwater protection strategies against further pollution (Balamurugan et al., 2020d; Johnny et al., 2018; Muniraj et al., 2021; Panneerselvam et al., 2021a, b). Recently, both natural and artificial sources of groundwater contamination have caused human health issues, which is the most critical problem in arid and semiarid regions worldwide (Balamurugan et al., 2020a; Patil et al., 2020). Moreover, it is essential to monitor the yield and quality of grains since contaminated water affects soil properties and natural ecosystem conditions (Balamurugan et al., 2020b).

Among the many contaminants, nitrate contamination is a critical issue to monitor, and its impact on human health must be evaluated. Geogenic and anthropogenic activities are primary sources of nitrate contamination of groundwater worldwide, i.e., in the USA, Europe, and India. Nitrate is highly soluble in water and quickly diffuses in groundwater (Nhu et al., 2020). In the southern part of India, rural and local residents directly draw water from boreholes for drinking and agricultural purposes. The continuous consumption of groundwater containing a high nitrate concentration has seriously increased health issues. In recent years, numerous studies (Adimalla, 2019) have been performed regarding the impact of nitrate contamination on drinking water and environmental issues. An elevated nitrate concentration in drinking water could cause liver damage, infant blue baby syndrome, and various forms of cancer (Kaur et al., 2020; Taneja et al., 2019).

Adimalla (2020) carried out a study on nitrate contamination of drinking water in the semiarid region of South India and found that agricultural activities and animal waste disposal are significant factors causing groundwater quality deterioration in the study region. In a semiarid region of India, Karunanidhi et al. (2019) investigated the potential health risk in hard rock regions due to fluoride and nitrate contamination. Contamination was also attributed to the excess use of synthetic fertilizers. Additionally, animal excrement applied as fertilizer in agricultural fields constituted another source of groundwater contamination. Tian et al. (2019) performed a risk assessment of nitrate-contaminated shallow groundwater in Changchun New District, China. It was reported that waste disposal in residential areas and modern agricultural activities are major sources of nitrate contamination in groundwater. Wagh, Panaskar, et al. (2020) conducted a risk assessment study of groundwater exhibiting severe nitrate contamination in the Kadava River basin in India. They found that waste disposal in residential areas near the river basin and modern agriculture activities greatly deteriorated the groundwater quality. Shukla and Saxena (2020a, b) reviewed nitrate contamination sources and leaching into groundwater. They determined that isotopic studies with the help of statistical tools could yield better results in nitrate contamination determination and identification than could geogenic factor studies. Nadikatla et al. (2020) evaluated the groundwater quality with the water quality index method in Srikakulam district, Andhra Pradesh, India. They found that the groundwater quality was adversely affected due to a lack of proper sanitation facilities, sewage disposal, and seepage runoff.

The water quality index (WQI) is the most powerful tool to estimate the drinking water quality in rural, urban, and industrial regions. The WQI expresses the groundwater quality in terms of values and reveals the status of contaminants in groundwater. In addition, statistical analysis has been employed to identify the major ions contributing to groundwater quality deterioration in the study region. In this domain, the correlation coefficient, principal component analysis (PCA), and hierarchical cluster analysis (HCA) are the most efficient methods to evaluate the chemical composition of groundwater. This facilitates the identification of the chemical composition of groundwater impacted by the processes of rock–water interaction, sewage intrusion, and evaporation during post-monsoon periods in arid and semiarid regions (Sajil Kumar, 2020; Singh et al., 2020; Su, Geng, et al., 2020).

Considering the above context, the specific objectives of the present study are (1) to evaluate the physiochemical characteristics of groundwater and compare these characteristics to World Health Organization (WHO) and Bureau of Indian Standards (BIS) requirements, (2) to determine the nitrate contamination level in the study area, (3) to ascertain the vulnerable regions based on the WQI value, and (4) to identify contamination sources via statistical analysis. The results of the present study could be useful for the development of practical approaches to enhance rural drinking water systems in nitrate-prone regions.

## Material and methodology

### Study area

Palani is a well-known religious touristic area in the southern part of India and a prominent taluk of Dindigul district. Palani lies between 10°20'–10°45^'^ N latitude and 77°20′0″ to 77°35′0″ E longitude and covers an area of 666.95 km^2^, excluding hilly terrain. The study boundaries encompass Coimbatore in the southeast and Madurai in the northwest. A subdivision of the Western Ghats borders the background of the town, namely the Palani Hills, where the slope station of Kodaikanal is located. The view from within the town is ruled by two slopes, i.e., Sivagiri and Sakthigiri. A sanctuary is located on the former slope. At the foot of these slopes occur a few lakes draining into the Shanmuga River, a tributary of the Amaravathi River. This river originates in the inclines of the Palani Hills. The study area, which is located at an elevation of 315 m above the mean sea level (MSL), exhibits an average annual temperature of 27.2 °C, and the annual rainfall reaches 630 mm. The highest temperature of approximately 29.6 °C is recorded in April and May.

### Geological setting

**Fig. 1:**
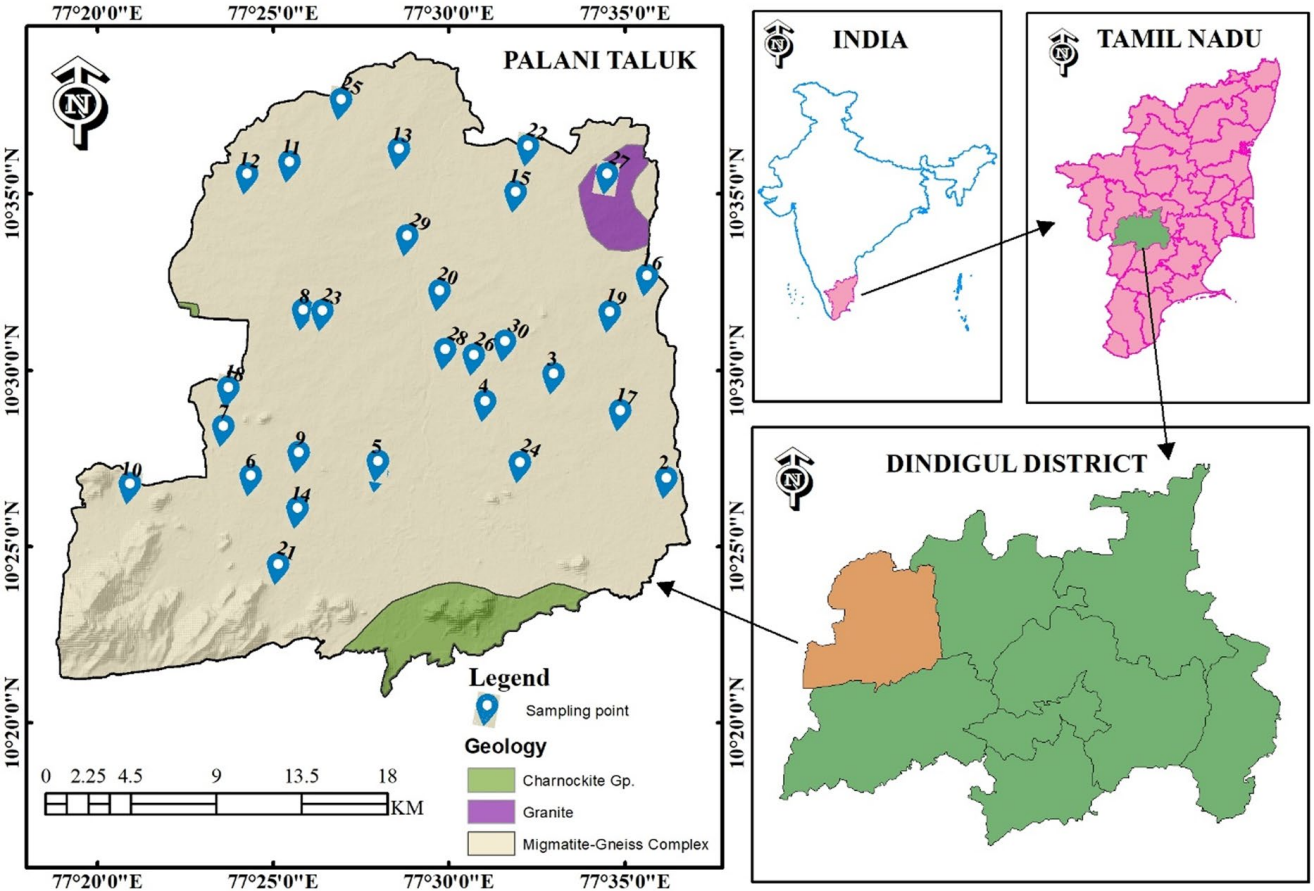
Sampling locations with geology of the study area

Hard rocks cover more than 95% of the study area. The gneissic rock type is the parent rock commonly encountered in the entire study area. Charnockite rock covers the southern part of the study area (Fig. 1). The observed pyroxene granulite is gray, and granulitic rock with minerals occurs on the weathered surface. This rock may consist of diopside, hypersthene, plagioclase, hornblende, biotite, and quartz (GSI, 1995). The elevation of the Palani hills ranges from 1163 to 2502 m, and the Sirumalai hill is located in the southeastern part of the study area. Plains are dominant between these hill ranges. Red soil and black cotton soil are frequently observed soil types in the study region. The soil geotechnical properties in the study area indicate high-permeability and fractured rock types.

### Sampling and analysis

Thirty sample locations were identified based on the high population density, agricultural activities, and waste disposal sites in the study area. The samples were collected in prewashed polyethylene bottles and subjected to preservation techniques to improve the accuracy of the results. Physical characteristics such as the pH, electrical conductivity, and total dissolved solids were measured during sample collection with pH, EC, and TDS meters, respectively (Hanna, HI-98129). Calcium and magnesium were determined via complexometric titration with ethylenediaminetetraacetic acid and disodium salt (EDTA) solutions. Sodium and potassium were determined with a flame photometry instrument (model S-931). Chloride was determined via Mohr’s method, and carbonate and bicarbonate were estimated with the titration method according to the APHA (APHA 2005). Sulfate and nitrate were estimated through UV–visible spectroscopy (LMSP UV1000B). Fluoride ions were determined via potentiometric analysis with a fluoride ion-selective electrode at 25 °C.

### Quality assurance and quality control

Quality assurance and quality control are efficient methods to obtain more accurate results during groundwater sampling and testing. One of the most important activities associated with sampling site selection satisfies the objective of the present research. Once suitably selected, sampling documentation and collection were carried out. All samples were collected in 1-l polyethylene bottles prewashed with a 10% HNO_3_ solution and distilled water. An ionic balance error (IBE) equation was applied to obtain the accuracy of the analytical results between the concentrations of major cations and anions in milliequivalent per liter (meq/L) for all collected samples (Eq. 1). The IBE value should not exceed the acceptable limit of ± 10%.

1 $${\text{IBE}} = \frac{{\sum {\text{Cations}} - \sum {\text{Anions}}}}{{\sum {\text{Cations}} + \sum {\text{Anions}}}} \times 100$$

### Water Quality Index (WQI)

The WQI indicates the nature of water in terms of values representing the present conditions of water for any intended use (Kirubakaran et al., 2015). Index values were calculated, followed by (1) the selection and assignment of a weight (*w*_*i*_) value to each water quality parameter based on its importance in the overall quality for drinking purposes, (2) calculation of the relative weight (*W*_*i*_) with Eq. (2), (3) determination of the quality rating (*q*_*i*_) for each parameter with Eq. (3), and (4) calculation of the subindex (SI_*i*_) value and summation of the subindex values to estimate the overall quality of water.

2 $$W_{i} = \frac{{w_{i} }}{{\mathop \sum \nolimits_{i = 1}^{n} w_{i} }}$$

3 $$q_{i} = \frac{{C_{i} }}{{S_{i} }} \times 100$$

4 $${\text{SI}}_{i} = W_{i} \times q_{i}$$

5 $${\text{WQI}} = \sum {\text{SI}}_{i}$$

### Hydro-facies analysis

Piper (1944) proposed a graphical representation of analytical data to reveal the major ions influencing the groundwater nature. Open-source diagram software was employed to generate a Piper diagram. The diagram suggested two tertiary plots of cations and anions and one diamond shape summarizing both tertiary plots. This diagram was produced based on the cation and anion concentrations at chemical equilibrium in groundwater. The Piper diagram results indicated that alkaline components exceeded alkaline components. The other major contamination sources and problems should be resolved via exhaustive studies of the critical water quality at specific sample locations.

### Geochemical mechanics

Gibbs (1970) proposed a plot to identify groundwater geochemical characteristics and evolution. This plot has been widely applied in groundwater studies to establish the relationship between the water composition and effects of the aquifer lithology on the groundwater chemistry (Muniraj et al., 2019). This diagram provides the exact mechanism controlling the groundwater chemistry in the study region. Precipitation, evaporation, and rock–water interactions are the three distinct fields of this plot.

### Health Risk Assessment (HRA)

HRA is the most important process to measure human exposure to contaminants via the consumption of groundwater containing elevated concentrations of chemical components (Shukla & Saxena, 2020a, 2020b). This process considers major components such as exposure media, time, concentration, receptor exposure, and nature of the environment (Table 1). According to the International Agency for Research on Cancer (IARC, 1965), elevated concentrations of nitrate can be categorized as posing a noncarcinogenic risk (https://www.iarc.who.int/). The present study considered the health effects of nitrate contamination through drinking water intake on residents of the high-density study area. The impact of a high nitrate concentration on the human body was calculated in two steps (Soleimani et al., 2020). In the first step, the chronic daily intake was estimated (Eq. 6), and in the second step, the hazard quotient was calculated based on the reference dose (Eq. 7):

6 $${\text{CDI}} = \frac{{{\text{CPW}} \times {\text{IR}} \times {\text{ED}} \times {\text{EF}}}}{{{\text{ABW}} \times {\text{AET}}}}$$

7 $${\text{HQ}} = \frac{{{\text{CDI}}}}{{{\text{RfD}}}}$$

where *E* is the chronic daily intake in mg/kg/day; CPW is the concentration of a specific pollutant in groundwater in mg/day; IR is the rate of human ingestion in l/day; ED is the duration of exposure in years; EF is the frequency of exposure in number of days/year; ABW is the average body weight in kg; AET is the average time in days; HQ is the noncarcinogenic hazard quotient; and RfD is the reference dose of nitrate in mg/kg/day (USEPA, 2004).

**Table 1:** USEPA (2004) standards for nitrate contamination

Parameter	Children	Male	Women
IR (L/Day)	0.78	2.5	2.5
ED (Years)	12	64	67
EF (Days/Years)	365	365	365
ABW (Kg)	15	65	55
AET (Days)	4380	23,360	24,455

### Statistical analysis

Statistical analysis of groundwater quality parameters promotes the identification of the reaction, interrelationship, and grouping of chemical components in the study region. The correlation analysis, principal component analysis (PCA), and hierarchical cluster analysis (HCA) techniques were implemented in the present study. These techniques are highly efficient tools for data classification and useful visualization methods to identify the source of contamination in groundwater (Egbueri, 2020; Marín Celestino et al., 2018). SPSS version 21.0 was employed to conduct statistical analysis of groundwater in the study area.

### Spatial analysis

Inverse distance weighted (IDW) interpolation is an effective method to determine cell values through linearly weighted combination of a set of sample locations in the study region (Panneerselvam, Karuppannan, et al., 2020). The surface of the study region was interpolated based on the latitude and longitude of the sample locations (Anand et al. 2020). The weight is a function of the inverse distance and was assigned to each location based on the distance. The concentration of each water quality parameter was visualized in spatial maps in ArcGIS (v10.4).

## Results and discussion

### General hydrochemistry

Statistical analysis results for the groundwater parameters in the study region are presented in Table 2. The concentration of hydrogen ions (pH) in water is very important. This helps identify the nature of groundwater, either acidic or alkaline (Balamurugan et al., 2020; Balamurugan et al., 2020; Balamurugan Kumar, & Shankar, 2020a; Balamurugan, Shunmugapriya, et al., 2020; Su, Wu, et al., 2020). However, the pH of groundwater in the study area ranged from 7.3 to 8.2, with an average value of 7.8. It is clear that EC is the most important parameter to estimate the total ionic concentration in groundwater, and an elevated EC value reflects the total dissolved solids in groundwater (Azhdarpoor et al., 2019). The EC value ranged from 650 to 6330 µS/cm in the study area with a mean value of 2236.5 µS/cm. The measured elevated EC value indicates that the nature of the aquifer, rock–water interactions, and anthropogenic activities highly influence the groundwater characteristics. Total dissolved solids (TDS) comprise major ions such as calcium, magnesium, sodium, potassium, chloride, sulfate, nitrate, carbonate, and bicarbonate dissolved in groundwater (Paul et al., 2019). TDS ranged from 358 to 3716 mg/L, with an average value of 1316.55 mg/L in the study area. A high TDS value could cause major health issues such as kidney stones, heart diseases, and stomach problems. TH is mainly attributed to the concentration of calcium and magnesium in groundwater (Marko et al., 2014). TH varied between 240 and 1600 mg/L in the study area, with a mean CaCO_3_ concentration of 653.16 mg/L.

**Table 2:** Descriptive statistical analysis of groundwater in the study area

Parameter	Min	Max	Avg	SD	WHO (2011)
pH	7.30	8.20	7.80	0.26	6.5–8.5
EC	650	6330	2236.5	1458.77	1500
TDS	358	3716	1316.5	875.94	500
TH	240	1600	653.17	384.04	200
Na^+^	12	122	51.40	28.84	200
Ca^2+^	20	400	135.93	88.60	200
Mg^2+^	6.08	194.4	76.14	46.52	150
K^+^	2	18	9.67	4.25	12
HCO_3_^−^	195.2	732	410.35	128.52	–
Cl^−^	25	893	282.73	221.88	200
SO_4_^2−^	15	322	100.02	90.59	200
NO_3_^−^	5	73	34.17	21.81	50
F^−^	0.12	1.79	0.58	0.41	1.5

**Fig. 2:**
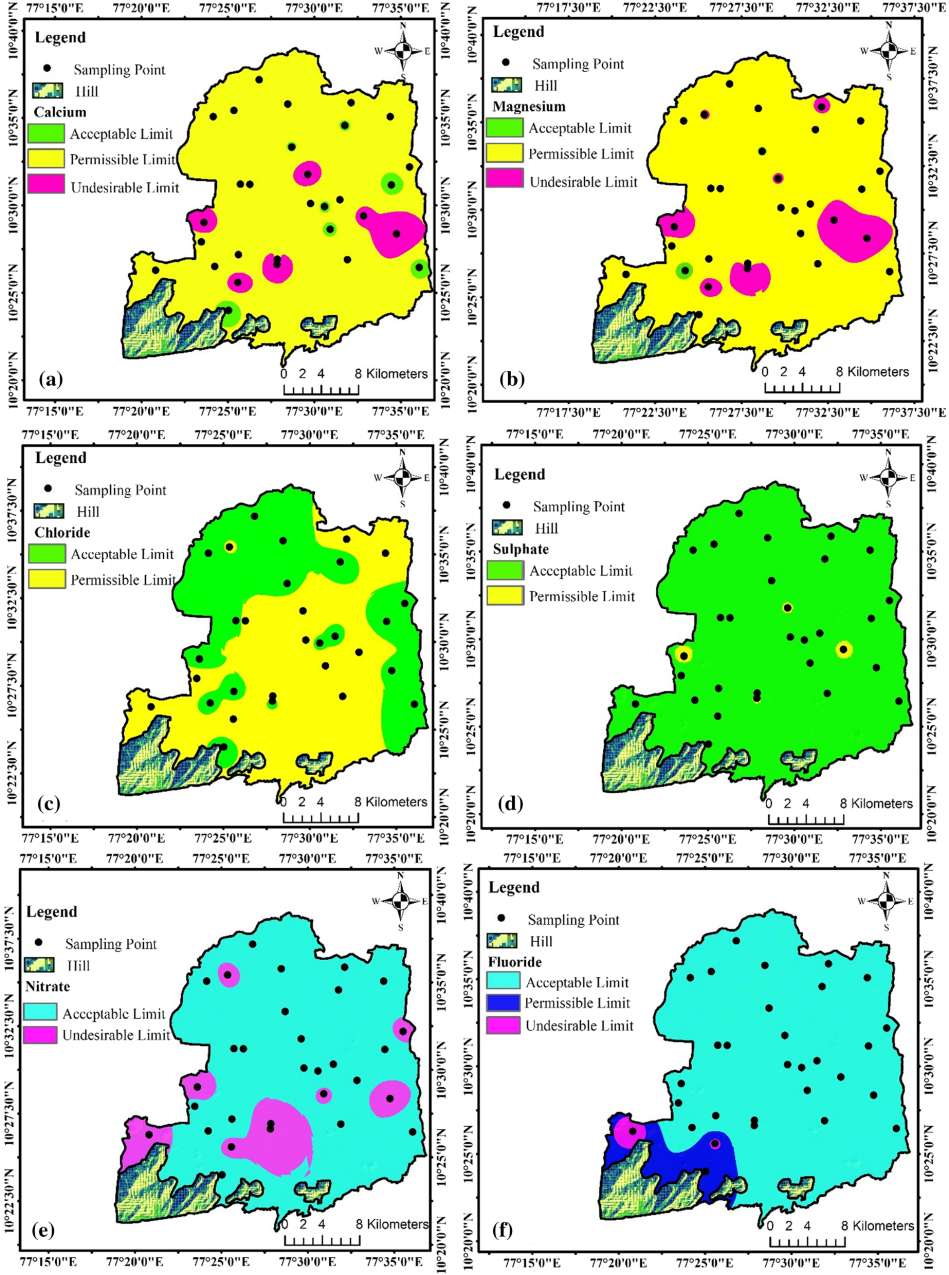
Spatial analysis of **a** calcium **b** magnesium **c** chloride **d** sulfate **e** nitrate and **f** fluoride

The order of cation dominance in the study area was as follows: calcium > magnesium > potassium > sodium. In the study area, sodium ranged from 12 to 122 mg/L, with an average concentration of 51.4 mg/L. All the sample locations remained below the permissible level of sodium for drinking purposes. Calcium and magnesium are major cations highly influencing the quality of groundwater. In the study area, calcium ranged from 20 to 400 mg/L, with an average concentration of 135.93 mg/L. In spatial analysis, a 16.97-km^2^ area reached the acceptable limit, 599.58 km^2^ reached the permissible limit, and 50.40 km^2^ (Fig. 2a) reached the desirable limit recommended by the WHO (2011). The magnesium concentration ranged from 6.07 to 194.4 mg/L, with a mean concentration of 76.14 mg/L. Approximately 3.22 km^2^ reached the acceptable limit, 599.46 km^2^ reached the permissible limit, and 64.27 km^2^ (Fig. 2b) was recorded as reaching the undesirable limit based on WHO standards. Excess concentrations of calcium and magnesium could cause severe health issues among humans involving water use for irrigation purposes (Aravinthasamy et al., 2019). Potassium ranged from 2 to 18 mg/L with a mean concentration of 9.66 mg/L, and 26.67% of the sample locations was highly affected due to high concentrations. Feldspar, microcline, orthoclase, and biotite weathering constituted the major mineral cause of the excess potassium concentration in groundwater. In the study area, the major dominance of anions exhibited the order of nitrate > chloride > sulfate > bicarbonate > fluoride > carbonate. The chloride concentration ranged from 25 to 893 mg/L, with a mean concentration of 282.73 mg/L. Spatial analysis revealed that 268.29 km^2^ was acceptable and 398.66 km^2^ (Fig. 2c) was permissible based on WHO standards (2011). The sulfate concentration varied between 15 and 322 mg/L, with an average concentration of 100.01 mg/L, and 659.94 km^2^ (Fig. 2d) reached the acceptable limit for drinking purposes. Both chloride and sulfate ions in the study region were observed to remain below the permissible limit, which did not affect the nature of groundwater for drinking purposes. Additionally, the bicarbonate concentration ranged from 195.2 to 732 mg/L, with an average concentration of 41.35 mg/L. The observed high bicarbonate concentration was attributed to the weathering of rock and rock–water interactions. However, nitrate is the most significant groundwater pollutant considering both drinking and agricultural uses. The nitrate concentration ranged from 5 to 73 mg/L, with a mean concentration of 34.16 mg/L. One-third of the sample locations was highly affected due to a high nitrate concentration. In the study area, dumping of waste in open land by residents, sewage disposal, and chemical fertilizers were the major factors causing nitrate contamination of groundwater. Spatial analysis revealed that 101.14 km^2^ (Fig. 2e) was undesirable for drinking purposes. Continuous consumption of nitrate-contaminated water could result in major diseases such as heart problems and blue baby syndrome (Kumar & Balamurugan, 2018; Panneerselvam, Paramasivam, et al., 2020). The fluoride ion concentration ranged from 0.12 to 1.79 mg/L, with an average concentration of 0.58 mg/L. Approximately 10% of the sample locations (9.05 km^2^) exceeded the permissible limit of fluoride for drinking purposes (Fig. 2f).

### Piper diagram

**Fig. 3:**
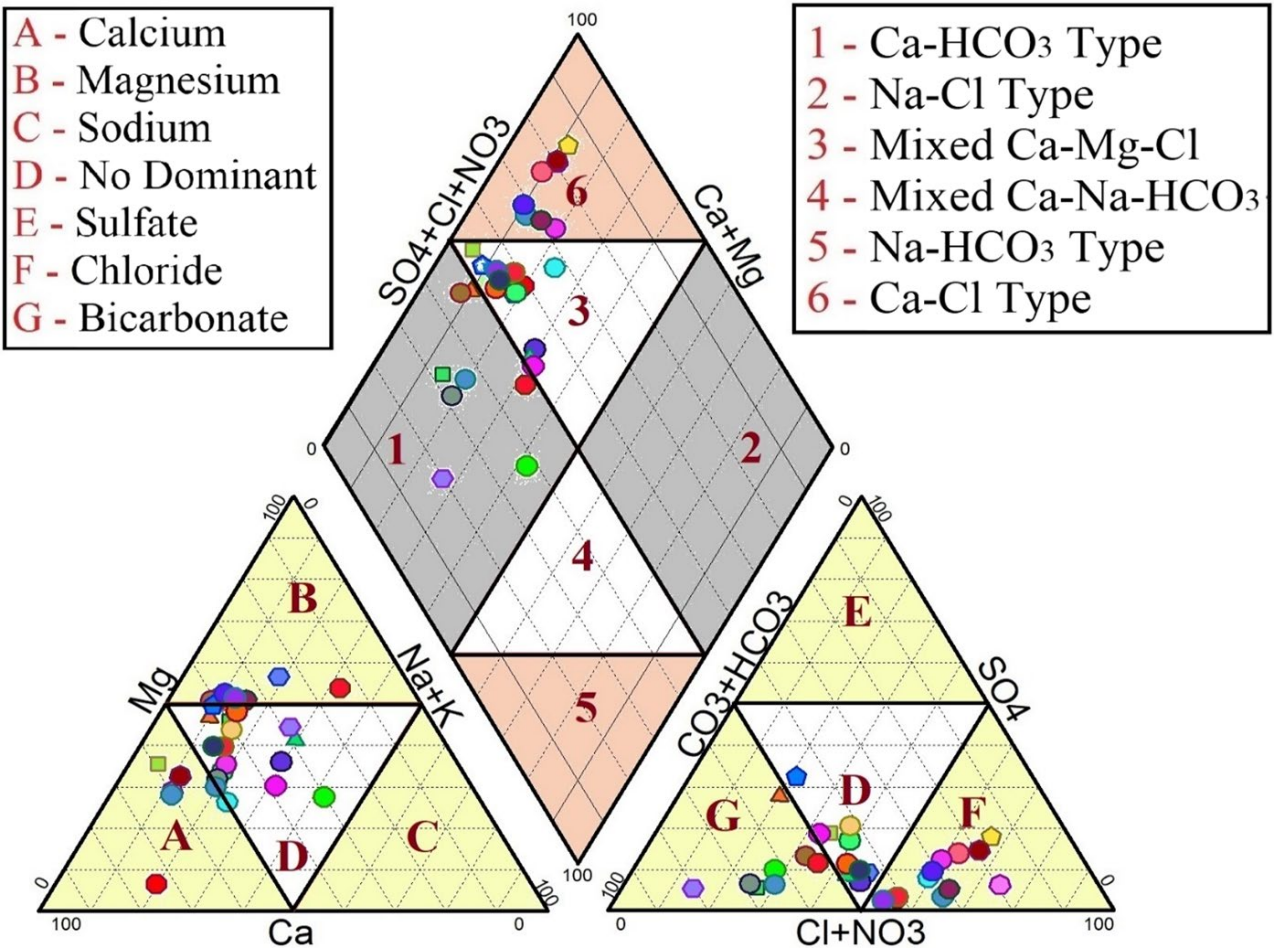
Piper trilinear diagram of the study area

A Piper plot for the tested groundwater in the study area is shown in Fig. 3. This figure reveals that certain sample locations exhibit mixed Ca^2+^–Mg^2+^–Cl^−^ effects, 26.66% of the sample locations exhibited Ca^2+^–Cl^−^ impacts, and 23.34% of the sample locations exhibited Ca^2+^–HCO_3_^−^effects. These results indicate that alkaline earth metals, weathering, rock–water interactions, inadequate rainfall, evaporation, and anthropogenic activities were the major factors influencing the characteristics of groundwater in the study area. Major ions, such as Ca^2+^, Mg^2+^, Cl^−^ and HCO_3_^−^, were highly dominant in terms of the groundwater quality.

### Gibbs plot

**Fig. 4:**
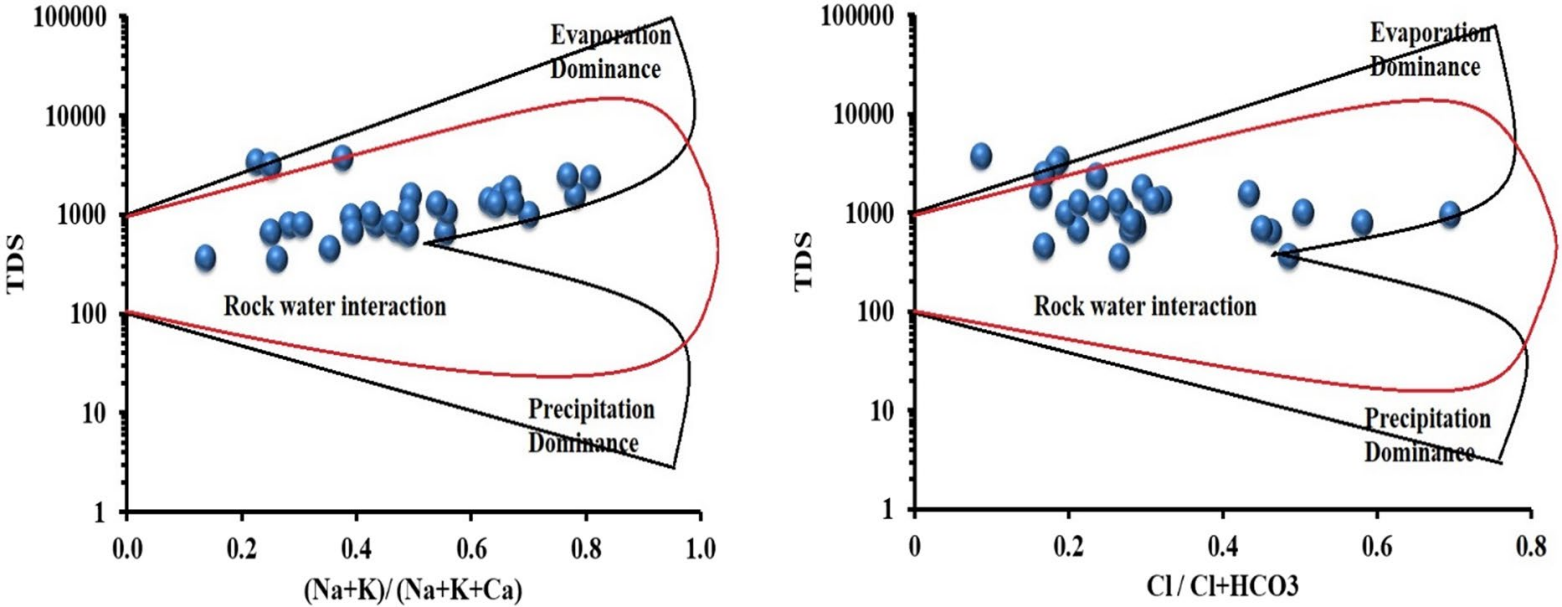
Gibbs plot of the study area

**Fig. 5:**
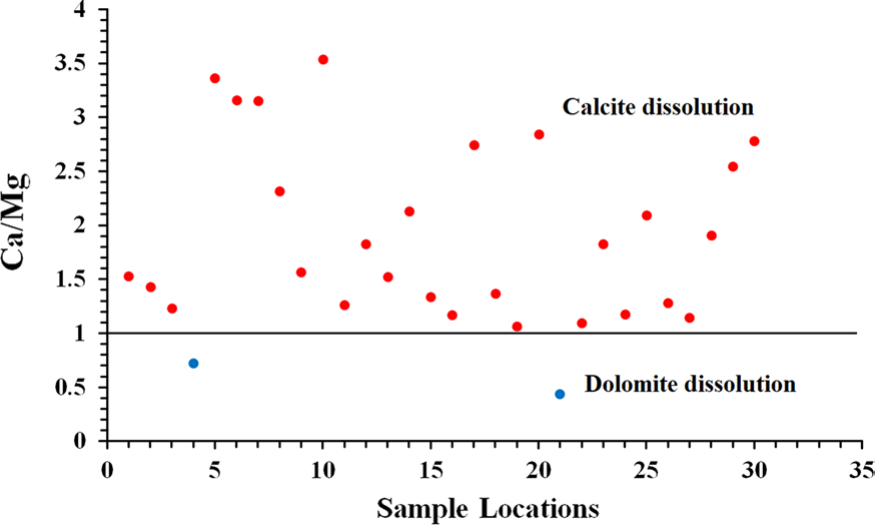
Molar ratio of Ca–Mg indicating dissolution of groundwater

The groundwater chemistry and evolution mechanisms in the study area are shown in Fig. 4. The Gibbs plot for the study area shows that 90% of the sample locations was highly influenced by rock–water interactions, and the remaining 10% of the sample locations was dominated by evaporation. The increasing impacts of parent rock weathering, oxidation–reduction, ion exchange, and mineral dissolution, such as calcite and dolomite dissolution, constituted the major factors resulting in groundwater quality deterioration (Panneerselvam et al., 2021a, b). Twenty-eight samples revealed calcite dissolution, and two samples fall indicated dolomite dissolution (Fig. 5).

### Water Quality Index (WQI)

**Fig. 6:**
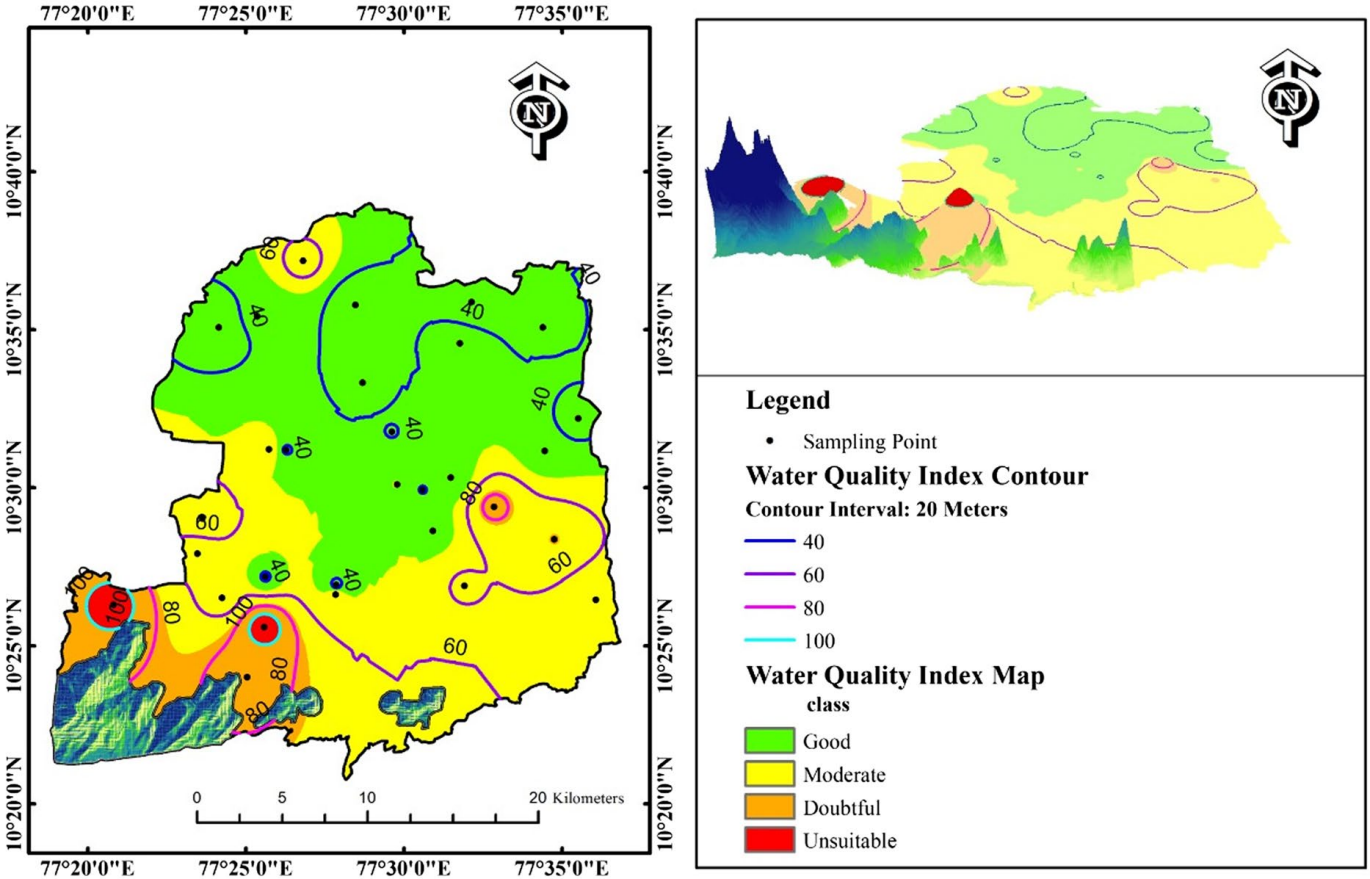
WQI map of the study region

The WQI was calculated to assess the present conditions of groundwater for drinking purposes in the study region. Groundwater classification based on the WQI value requires that a value less than 25 indicates an excellent quality, 26–50 indicates a good quality, 51–75 indicates a moderate quality, 76–100 indicates a doubtful quality, and a value greater than 100 indicates unsuitable conditions for drinking water purposes. In the study area, the WQI value ranged from 28.70 to 117.15, with an average value of 53.59. Approximately 53.33% of the sample locations exhibit good conditions, 26.67% of the sample locations exhibited moderate conditions, 13.33% of the sample locations indicated doubtful conditions, and 6.67% of the sample locations (2 locations) indicated unsuitable water conditions (Table 3). The highly contaminated zones were visualized through 3D spatial analysis (contour lines) (Fig. 6). These results indicate that high values of the WQI were recorded along the footpath in hilly terrain. A more significant amount of waste disposal from households, sewage disposal, excess utilization of fertilizers, and pesticides for agriculture are the major factors influencing groundwater quality for drinking purposes.

**Table 3:** WQI classification of groundwater in the study area

WQI value	Water class	No. of samples	% of samples	Remarks
0–25	Excellent	0	0.00	Fit for drinking and irrigation uses
26–50	Good	16	53.33	
51–75	Moderate	8	26.67	Treatment needed for drinking and fit for irrigation uses
76–100	Doubtful	4	13.33	Irrigation uses
> 100	Unsuitable	2	6.67	Need to treat for irrigation uses

### Human health risk assessment

**Fig. 7:**
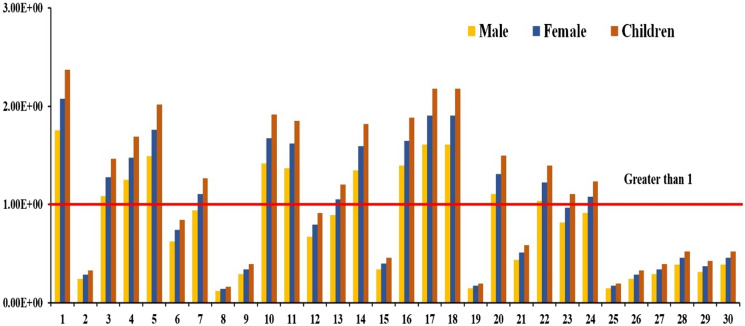
Hazards index value in the study area

Nitrate (NO_3_^−^) is one of the world's largest sources of groundwater contamination. The specific objective of this study was to assess the impact of the continuous consumption of highly nitrate-contaminated water on human health. High concentrations of nitrate could result in adverse health effects, categorized as noncarcinogenic risks to humans (Gao et al., 2020). In the study area, the hazard index value for men ranged from 1.20E−01 to 1.75E + 00 (with an average value of 8.21E−1), that for women from ranged from 1.42E−01 to 2.07E + 00 (with a mean value of 9.71E−01), and that for children ranged from 1.63E−01 to 2.37E + 00 (with a mean value of 1.11E + 00). At approximately 40, 50, and 53.33% of the sample locations, the HQ value exceeded the recommended values for men, women, and children, respectively (Fig. 7). This indicates that children and women occurred at a higher risk than did men through drinking water ingestion. However, groundwater is the major source of drinking water. Notably, children face adverse health risks through the intake of contaminated groundwater. The major sources of the observed elevated nitrate concentrations in the study area included the excessive use of fertilizer, pesticide leaching from waste dumping, and sewage disposal.

### Statistical analysis

#### Correlation analysis

Pearson's correlation matrix has been widely applied to identify the role of water quality parameters and the impact on groundwater chemistry deterioration. The *R* value typically ranges from −1 to + 1, with a positive value indicating a high correlation, and a negative value indicating a low correlation (Wagh, Mukate, et al., 2020). In the study area, correlation coefficient analysis (Table 4) revealed that the pH value attained a negative correlation with EC, TDS, TH, Na^+^, Ca^2+^, Mg^2+^, K^+^ Cl^−^, SO_4_^2−^, NO_3_^−^ and F^−^ and a positive correlation with CO_3_^2−^ (*r*^2^ = 0.31) and HCO_3_^−^ (*r*^2^ = 0.11), which indicates a moderate correlation with each of the remaining parameters. EC achieved a high positive correlation with TDS (*r*^2^ = 1.00), TH (*r*^2^ = 0.93), Ca^2+^ (*r*^2^ = 0.88), Mg^2+^ (*r*^2^ = 0.85), SO_4_^2−^ (*r*^2^ = 0.80) and NO_3_^−^ (*r*^2^ = 0.74) and a low positive correlation with Na^+^ (*r*^2^ = 0.17), K^+^ (*r*^2^ = 0.32), HCO_3_^−^ (*r*^2^ = 0.59) and Cl^−^ (*r*^2^ = 0.36). TDS exhibited a positive correlation with TH, Ca^2+^, Mg^2+^, SO_4_^2−^ and NO_3_^−^ and a negative correlation with CO_3_^2−^ (*r*^2^ = 0.33). TH attained a high positive correlation with Ca^2+^ (*r*^2^ = 0.94), Mg^2+^ (*r*^2^ = 0.92) and SO_4_^2−^ (*r*^2^ = 0.83) and a negative correlation with CO_3_^2−^ (*r*^2^ = 0.29). This indicates that the reverse ion exchange and weathering processes dominated the nature of groundwater. Since NO_3_^−^ was positively correlated with Ca^2+^ (*r*^2^ = 0.70) and Mg^2+^ (*r*^2^ = 0.69), agricultural activities highly influenced the characteristics of groundwater. SO_4_^2−^ attained a high positive correlation with Mg^2+^ (*r*^2^ = 0.86), indicating anthropogenic influences.

**Table 4:** Correlation analysis of water quality parameters

	pH	EC	TDS	TH	Na^+^	Ca^2+^	Mg^2+^	K^+^	CO_3_^2−^	HCO_3_^−^	Cl^−^	**SO**_**4**_^**2−**^	**NO**_**3**_^**−**^	**F**^**−**^
pH	1.00	−0.41	−0.42	−0.48	−0.06	−0.50	−0.37	−0.03	0.31	0.11	−0.28	−0.30	−0.67	−0.14
EC		1.00	**1.00**	**0.93**	0.17	**0.88**	**0.85**	0.32	−0.32	0.59	0.36	**0.80**	0.74	0.06
TDS			1.00	**0.93**	0.17	**0.88**	**0.85**	0.32	−0.33	0.59	0.37	**0.80**	0.77	0.05
TH				1.00	0.28	**0.94**	**0.92**	0.30	−0.29	0.38	0.48	**0.83**	0.74	0.07
Na^+^					1.00	0.25	0.26	0.03	−0.27	0.22	0.21	0.30	0.23	0.09
Ca^2+^						1.00	0.73	0.29	−0.30	0.31	0.48	0.70	0.70	−0.18
Mg^2+^							1.00	0.27	−0.23	0.41	0.42	**0.86**	0.69	−0.01
K^+^								1.00	0.04	0.16	0.48	0.25	0.27	0.08
CO_3_^2−^									1.00	−0.38	−0.33	−0.27	−0.36	−0.03
HCO_3_^−^										1.00	0.10	0.42	0.31	0.00
Cl^−^											1.00	0.41	0.38	0.12
SO_4_^2−^												1.00	0.55	0.04
NO_3_^−^													1.00	0.04
F^−^														1.00

Higher positive correlate values are highlighted in bold

### Principal component analysis (PCA)

**Fig. 8:**
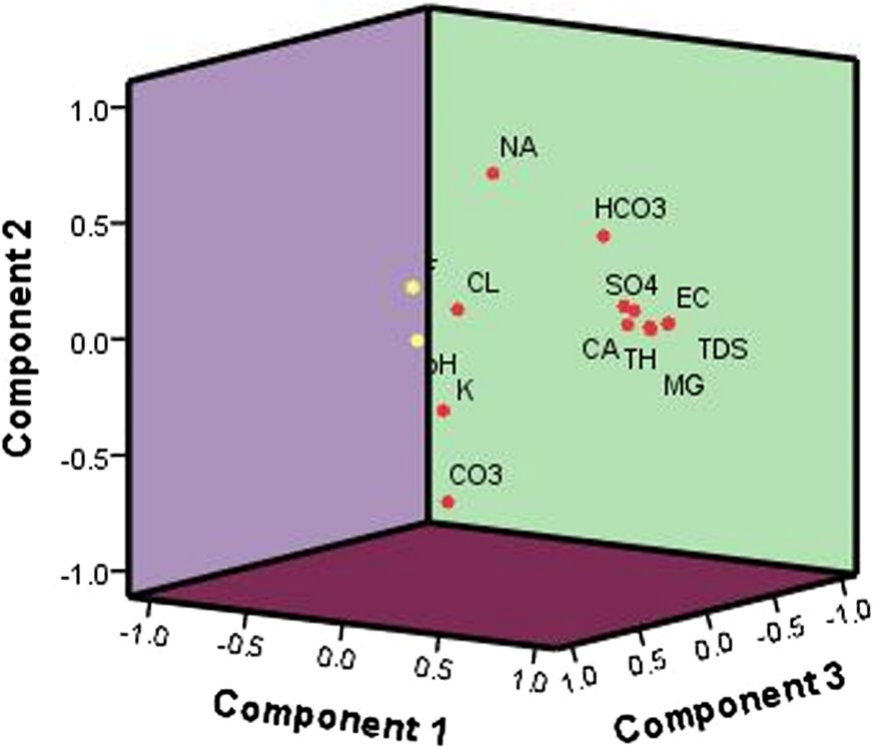
Factor component plot in rotated space

The PCA results demonstrated that primary water quality parameters, such as the calcium, magnesium, sulfate, and nitrate concentrations, declined due to human activities in the study region. The varimax method was adopted to rotate the parameters in PCA and extraction via the exclusion of eigenvalues higher than one (Pande et al., 2019). In the study region, PCA illustrated four factors responsible for the data structure (Table 5), accounting for 76.635% of the total variance and significant component values are in bold. Factor 1 comprised 49.902% of the total variance with high loadings for EC, TDS, TH, Ca^2+^, Mg^2+^, SO_4_^2−^ and NO_3_^−^ (Fig. 8). This result indicates that anthropogenic activities, such as the disposal of waste by residents, sewage intrusion, and chemical synthetic fertilizers application to obtain high crop yields, were the major reasons for the excess concentrations of salt and other ions.

**Table 5:** Component value of water quality parameters

Parameter	Component			
	1	2	3	4
pH	−0.519	**0.658**	0.318	0.261
EC	**0.953**	0.149	0.079	−0.147
TDS	**0.958**	0.137	0.070	−0.151
TH	**0.967**	−0.025	0.069	−0.107
Na^+^	0.314	0.163	−0.413	0.497
Ca^2+^	**0.903**	−0.126	0.036	−0.076
Mg^2+^	**0.895**	0.097	0.096	−0.126
K^+^	0.363	−0.112	**0.675**	0.436
CO_3_^2−^	−0.417	−0.049	**0.634**	−0.304
HCO_3_^−^	0.504	**0.692**	−0.098	0.175
CL^−^	0.536	−0.333	0.176	**0.519**
SO_4_^2−^	**0.846**	0.163	0.069	−0.023
NO_3_^−^	**0.817**	−0.227	−0.136	−0.147
F^−^	0.091	−0.371	−0.066	0.418
Eigenvalue	6.986	1.347	1.223	1.173
Total % of variance	49.902	9.618	8.739	8.376
Cumulative % of variance	49.902	59.520	68.259	76.635

Factor 2 was responsible for 9.618% of the total variance, with high factor loadings for pH and HCO_3_^−^ and weak positive loadings for EC, TDS, Na^+^, Mg^2+^, and SO_4_^2−^. This suggests that the high value of hydrogen ions could be attributed to parent rock weathering, rock–water interactions, and ion exchange process. These factors are highly influencing factors of groundwater quality degradation. Factor 3 comprised a total variance of 8.739% with high factor loadings for K^+^ and CO_3_^2−^ and a weak positive loading for HCO_3_^−^. This indicates that the major reason was the weathering of potassium-rich minerals such as feldspars, calcite, and dolomite. In addition, easily soluble fertilizers containing 15.5% nitrogen and 18% calcium are applied in the area. The use of fertilizers allows initial plant growth intensification, but afterward, fertilizer residues are completely dissolved and contaminate groundwater. In regard to Factor 4, the total variance explained reached 8.376% with high sodium and chloride ion loadings. Due to the impact of the ion exchange process, rock–water interactions and anthropogenic activities highly affected the groundwater quality in the study region.

#### Hierarchical cluster analysis (HCA)

**Fig. 9:**
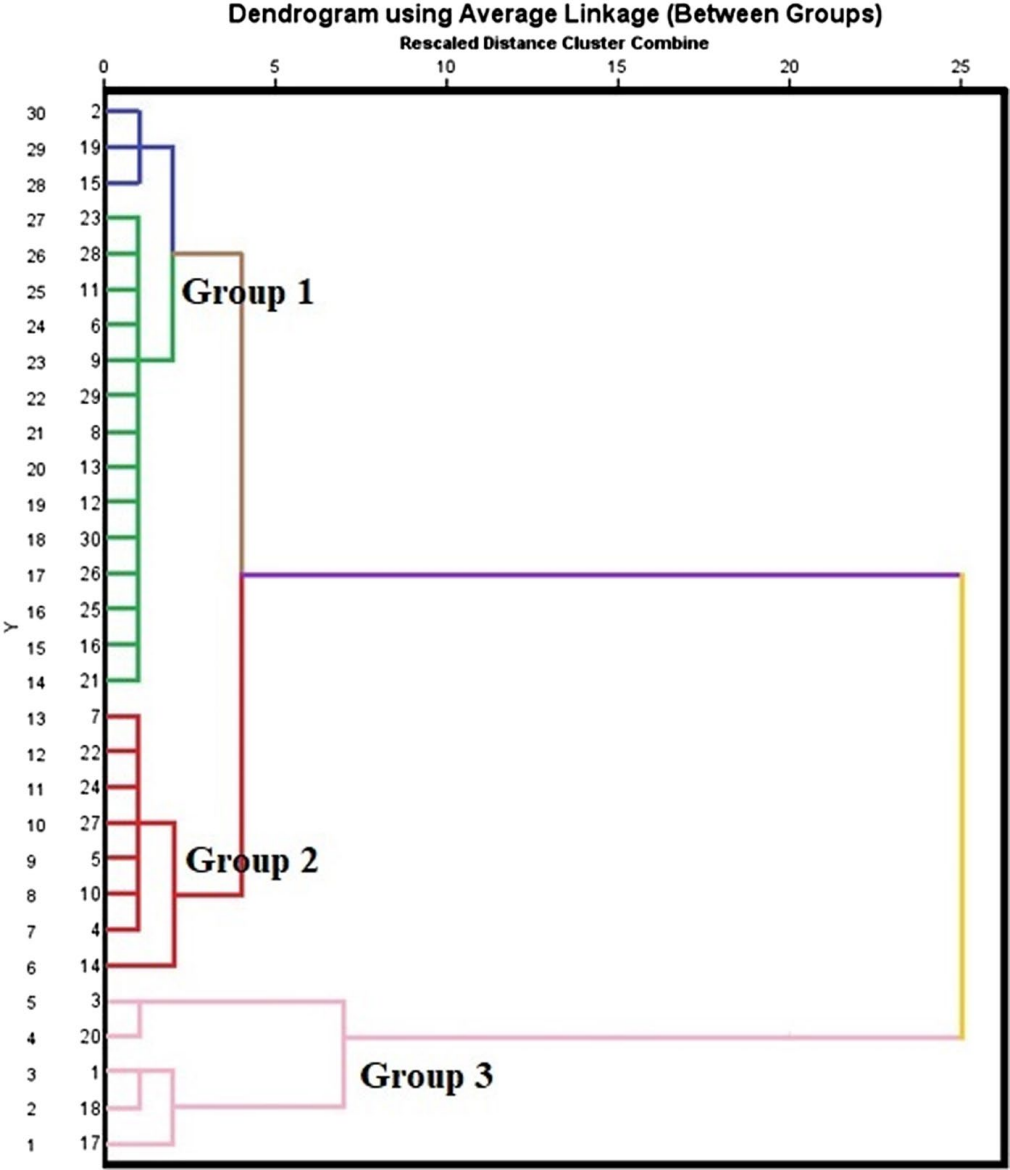
Dendrogram plot of average linkage between sample locations

The HCA results for the study area demonstrated that there were three groups. Group 1 comprised sample locations 1, 3, 17, 18 and 20, Group 2 encompassed sample locations 4, 5, 7, 10, 14, 22, 24 and 27, and Group 3 included sample locations 2, 6, 8, 9, 11, 12, 13, 15, 16, 19, 21, 23, 25, 26, 28, 29 and 30 (Table 6). Certain sample locations exhibited the same groundwater characteristics and less contamination (Fig. 9). The HCA results and grouped sample locations revealed that geogenic and reverse ion exchange processes and anthropogenic activities such as waste disposal, sewage intrusion, and excess utilization of synthetic fertilizers highly influenced the groundwater nature.

**Table 6:** Groups of groundwater sample in study area

Group	No. of groundwater parameter	No. of sample	% of samples
I	1, 3, 17, 18 and 20	5	16.67
II	4, 5, 7, 10, 14, 22, 24 and 27	8	26.66
III	2, 6, 8, 9, 11, 12, 13, 15, 16, 19, 21, 23, 25, 26, 28, 29 and 30	17	56.67

## Conclusion

This study aimed to increase the credibility of an integrated GIS and statistical methodology in groundwater health risk assessment of nitrate contamination and determination of the suitability of groundwater for drinking purposes.

- The Piper and Gibbs plots revealed that the processes of parent rock weathering, oxidation–reduction, ion exchange, and mineral dissolution, such as calcite and dolomite dissolution, were the major factors of groundwater quality deterioration.
- The water quality status for drinking purposes at approximately 26.67% of the sample locations was moderate, the water quality status was doubtful at 13.33% of the sample locations, and the water quality status was unsuitable at 6.67% of the sample locations (2 locations).
- The risk assessment results indicated that at 40, 50, and 53.33% of the sample locations, the HQ value exceeded the recommended values for men, women, and children, respectively. This indicates that children and women occurred at a higher risk than did men considering drinking water consumption.
- Statistical analysis in this study revealed that calcium, magnesium, chloride, and nitrate ions highly influenced the nature of groundwater. The study results capture the present nature and sources of contamination in the study area.

Local authorities and water resource managers should implement awareness programs to encourage farmers to adopt organic fertilizers instead of synthetic fertilizers. The integrated methodology in the present study reveals the current status of groundwater. This methodology satisfies the various indices with combined results to evaluate the suitability of groundwater in arid and semiarid regions worldwide. Additionally, this method can be applied to shallow aquifers, which are the primary sources of groundwater for both domestic and irrigation uses. It is very difficult to identify contaminated areas when viewed individually. Therefore, by conducting integrated studies, decision-makers could better understand the current state of groundwater purification and ensure that people only consume suitable water.

## Scope and recommendations

This work could help future researchers in human health risk assessment and artificial recharge management planning ensure sustainable and noncarcinogenic appraisal of the groundwater quality in arid and semiarid regions worldwide. This study recommends that proper waste disposal methods should be followed, sewage lines should be periodically inspected to avoid leakages, and awareness programs for farmers should be implemented highlighting the use of organic fertilizers instead of synthetic fertilizers.
